# Adult Bronchial Inflammatory Myofibroblastic Tumor: A Case Report

**DOI:** 10.2174/0115734056415231251020111548

**Published:** 2025-10-29

**Authors:** Zhi-Hui Zheng, Bo Shao, Li-Kang Luo, Jia-Cheng Guan

**Affiliations:** 1 Department of Radiology, The Second People’s Hospital of Quzhou, Quzhou 324000, Zhejiang Province, China; 2 Department of Radiology, Shulan (Quzhou) Hospital, Quzhou 324000, Zhejiang Province, China; 3 Department of Pathology, The Second People’s Hospital of Quzhou, Quzhou 324000, Zhejiang Province, China

**Keywords:** Inflammatory myofibroblastic tumor, Squamous cell carcinoma, Anaplastic lymphoma kinas, Computed tomography

## Abstract

**Introduction:**

Inflammatory myofibroblastic tumor (IMT) is a neoplasm originating from mesenchymal tissue and can occur in multiple parts of the body, such as the lungs, abdomen, pelvis, and retroperitoneum. Although the lung is a relatively common site for IMT, airway involvement in adults is rare, and most reported cases involve the central airway. Reports of IMT arising within the bronchus are uncommon.

**Case Presentation:**

We, herein, report the case of a 72-year-old male patient with bronchial IMT who was admitted due to a recurrent cough that worsened over two weeks. Tumor markers showed no significant elevation, and imaging examinations suggested a tumor in the left upper lobe bronchus. Due to the suspicion of malignancy, the patient underwent thoracoscopic left upper lobectomy. Postoperative pathological examination revealed an inflammatory myxoid myofibroblastic tumor of the left upper lobe bronchus. During a 12-month postoperative follow-up, no significant signs of metastasis or recurrence were observed.

**Conclusion:**

We have reported the case of endobronchial IMT in an adult, with a degree of contrast enhancement on CT lower than that previously reported for intratracheal IMT. The tumor lacks specific clinical symptoms and laboratory findings, which poses a challenge for accurate and timely preoperative diagnosis. Based on literature reports, in patients with recurrent cough, hemoptysis, or dyspnea, if CT shows a smoothly marginated endobronchial nodule with mild enhancement, the possibility of this disease should be considered.

## 1 INTRODUCTION

The histological origin and pathogenesis of IMT remain unclear. Clinical symptoms are non-specific, and patients with early-stage tracheal IMT are usually asymptomatic. As the tumor progresses, the most common symptoms are frequent wheezing and cough, often misdiagnosed as asthma. Therefore, IMT is prone to misdiagnosis and missed diagnosis. In view of cases of local recurrence and metastatic spread after surgical resection, as well as advances in cytogenetic analysis, inflammatory myofibroblastic tumor (IMT) is now classified as a tumor with low-grade malignant potential [1]. A recent study reported that the incidence of inflammatory myofibroblastic tumors is approximately 0.3% [2]. Inflammatory myofibroblastic tumor most commonly occurs in the lungs, accounting for approximately 0.04-0.07% of all lung tumors, and similar lesions can also be found in other parts of the body [3]. Inflammatory myofibroblastic tumors occurring in the trachea are rare, predominantly affecting patients under 16 years of age [4], with adult cases seldom reported. Tracheal IMT has a high preoperative misdiagnosis rate and is frequently misidentified as other malignant tracheal tumors. This paper reports a case of endobronchial IMT in a 72-year-old male patient and reviews the relevant literature.

## 2 CASE PRESENTATION

A 72-year-old male patient presented with a recurrent cough that persisted for two months and had worsened over the past two weeks. He denied symptoms, such as dyspnea, chest pain, fever, or hemoptysis. There was no significant past medical or family history. Physical examination, including nasopharyngeal assessment, and vital signs revealed no notable abnormalities. Laboratory tests, including complete blood count (CBC), C-reactive protein (CRP), and tumor markers, were all within normal limits.

**Fig. (1):**
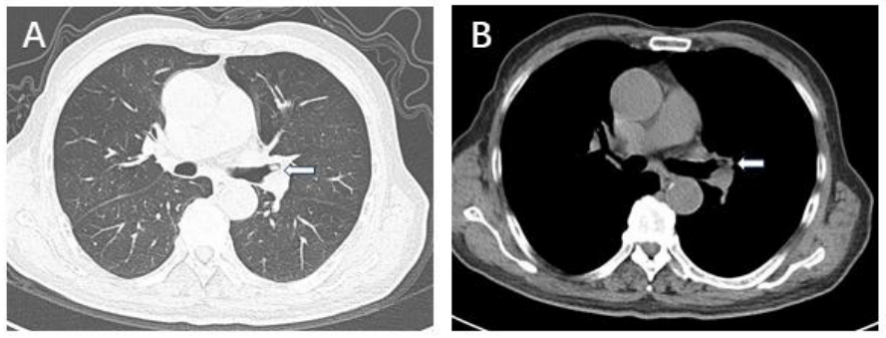
Medical image. **A:** Chest CT scan (lung window) shows a round-like nodule within the lumen of the left upper lobe bronchus (arrow). **B:** Mediastinal window shows the nodule within the left upper lobe bronchus (arrow).

**Fig. (2):**
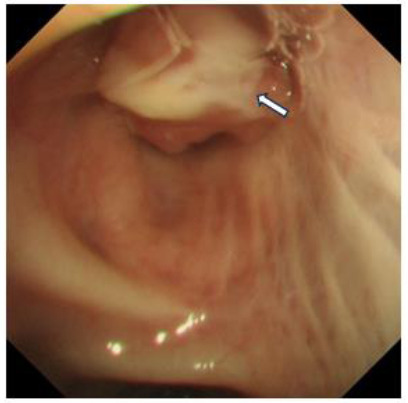
Bronchoscopic image. Bronchoscopy revealed a broad-based protruding mass in the left upper lobe bronchus (arrow).

**Fig. (3):**
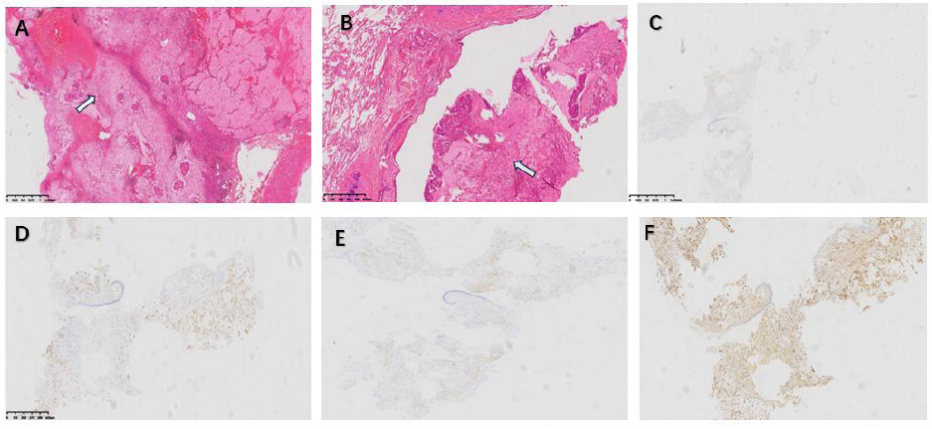
Histopathological image. **A:** Histology shows bronchial mucosal hyperplasia and a nodular proliferation of spindle cells in the submucosa(arrow). **B:** The stroma shows abundant mucoid material forming mucous lake-like changes, accompanied by infiltration of lymphocytes, plasma cells, and scattered neutrophils(arrow). **C:** Immunohistochemistry shows Actin(+). **D:** CD68(+). **E:** SMA(+). **F:** Vimentin(+).

Chest CT plain and contrast-enhanced scans in the lung window revealed a roundish nodule within the lumen of the left upper lobe bronchus (Fig. **1A**), while the mediastinal window showed the nodule inside the left upper lobe bronchus (Fig. **1B**). Bronchoscopy revealed a tumor in the left upper lobe bronchus (Fig. **2**). Due to concerns that bronchoscopic resection might lead to residual tumor, the patient consented to a thoracoscopic left upper lobectomy. Postoperative pathology revealed bronchial mucosal hyperplasia and nodular proliferation of spindle cell tumors in the submucosa (Fig. **3A**). The stroma showed abundant mucinous material forming mucin lake-like changes, with infiltration of lymphocytes, plasma cells, and a few neutrophils (Fig. **3B**). Immunohistochemistry was positive for actin, Cluster of differentiation 68 (CD68), Smooth Muscle Action (SMA), and vimentin (Figs. **3C**-**F**), confirming the diagnosis of inflammatory myofibroblastic tumor (IMT). At twelve months postoperatively, follow-up showed no obvious tumor recurrence.

## 3 DISCUSSION

IMT is a rare mesenchymal tumor and falls within the category of mesenchymal neoplasms. Tracheal IMT is uncommon. A review of cases in the literature showed that patients often present with symptoms, such as cough and dyspnea, but clinical manifestations lack specificity, and all tumors are located in the central airway. In three cases, preoperative-enhanced CT scans [4-6] revealed tumors with smooth morphology and significant enhancement. All patients underwent surgical resection, and no obvious recurrence was observed during follow-up of 9-12 months postoperatively, indicating a favorable prognosis.

Histopathologically, IMT typically exhibits three basic histologic patterns: (a) a myxoid/vascular pattern with spindle or stellate myofibroblasts in an abundant myxoid stroma accompanied by an inflammatory component; (b) compact spindle cells that may form a storiform pattern intermingled with inflammatory cells; and (c) dense plate-like collagen with low cellularity and a sparse inflammatory cell stroma. Immunohistochemically, IMT is characteristically positive for muscle-specific actin, smooth muscle actin, vimentin, and desmin [7].

Imaging studies are an important method for assessing tracheal tumors. On CT, IMT typically presents as an intratracheal mass lesion, characterized by a well-defined nodule with a regular shape and smooth margins. On contrast-enhanced CT scans, mild enhancement of the intratracheal mass can be observed. In this case, the patient's CT scan showed a nodule within the bronchial lumen of the left lower lobe, with a smooth lesion contour. Contrast-enhanced imaging demonstrated mild enhancement, which was lower than the enhancement levels previously reported for intratracheal IMTs. This difference was analyzed to possibly relate to the histological growth pattern; this case corresponded more closely to the type A growth pattern and was predominantly composed of myxoid components. The imaging findings were consistent with the histopathological features observed under the microscope. Intratracheal IMT needs to be differentiated from malignant tumors of the trachea.

Squamous cell carcinoma (SCC) is the most common primary malignant tumor of the trachea. SCC is closely associated with smoking, and CT findings typically include an irregularly marginated or lobulated tumor with an indistinct boundary from the tracheal wall, showing marked enhancement on contrast-enhanced scans [8].

Adenoid cystic carcinoma: It commonly occurs in the trachea and main bronchi. CT findings are primarily characterized by diffuse wall thickening, with mild to moderate and progressive enhancement. The lesion involves the tracheal wall more extensively than IMT [9]. The boundary with surrounding tissues, which may be indistinct, often becomes more clearly demarcated after CT enhancement, aiding in differentiation.

Mucoepidermoid carcinoma: It commonly occurs in lobar or segmental bronchi, presenting as an intraluminal oval nodule or mass, with margins that can be well-defined or ill-defined, and mild to moderate enhancement [10]. When it primarily exhibits intraluminal growth, differentiation from IMT is difficult.

For localized inflammatory myofibroblastic tumor (IMT), complete surgical resection is the treatment of choice, and postoperative prognosis is generally favorable in both adult and pediatric patients. Overall, pediatric IMT has a relatively good prognosis; even in cases that are unresectable at initial diagnosis or are anaplastic lymphoma kinase (ALK)-negative, favorable clinical outcomes are often achievable. Clinical observations have shown that regimens combining low-toxicity vinca alkaloids with low-dose methotrexate yield high response rates with good tolerability [11]. However, in adults with unresectable, recurrent, or metastatic disease, treatment strategies should be determined based on molecular subtype.

In the present case, molecular testing was not performed. In recent years, molecular pathological studies have identified a variety of gene fusions in IMT, including the frequently observed ALK rearrangement, as well as c-ros oncogene 1, receptor tyrosine kinase (ROS1), rearranged during transfection (RET), and neurotrophic tropomyosin receptor kinase (NTRK) 1/3 fusions [12]. These alterations provide potential targets for therapy.

In ALK-positive IMT, crizotinib is recommended as first-line therapy. For patients who develop resistance to crizotinib, treatment options include ceritinib, alectinib, or lorlatinib. In ROS1-, NTRK-, or RET-rearranged IMT, corresponding targeted agents, such as crizotinib, entrectinib, or selpercatinib, may be considered [13].

For ALK-negative IMT without actionable targets, chemotherapy remains the mainstay of treatment. Similar to other non-small-cell sarcomas, anthracycline-based regimens (doxorubicin ± ifosfamide) or methotrexate combined with vinca alkaloids achieve an objective response rate (ORR) of approximately 50%. The efficacy of anthracycline-based therapy is comparable to that of methotrexate-vinca alkaloid combinations [14].

Therefore, molecular profiling is recommended for all patients with IMT to enable accurate matching with targeted agents or optimal chemotherapy regimens, thereby achieving individualized treatment goals.

## CONCLUSION

In conclusion, adult bronchial IMT is a rare tumor. In this case, the IMT was located in the left bronchus, and its CT enhancement was lower than that of IMT in the central airways. It is often misdiagnosed as mucoepidermoid carcinoma. For mildly enhancing endobronchial nodules, bronchial inflammatory myofibroblastic tumor should be considered in the differential diagnosis.

## Data Availability

The data and supportive information are available within the article.

## LIST OF ABBREVIATIONS

IMT: Inflammatory myofibroblastic tumor
CT: Computed tomography
SMA: Smooth muscle actin
CD68: Cluster of differentiation 68
CRP: C-reactive protein
CBC: Complete blood count
SCC: Squamous cell carcinoma
ALK: Anaplastic lymphoma kinase
ROS1 c-ros oncogene 1: Receptor tyrosine kinase
RET: Rearranged during transfection
NTRK1/3: Neurotrophic tyrosine kinase receptor type 1 or 3
ORR: Objective response rate
NSCLC: Non-small-cell lung cancer

## STANDARDS OF REPORTING

CARE guidelines were followed.
